# Perceived stress and sleep quality in young and middle-aged patients with coronary heart disease: the mediating role of perceived social support and mental health literacy

**DOI:** 10.3389/fpsyg.2025.1444831

**Published:** 2025-05-29

**Authors:** Ying Lu, Juan Li, Zhenzhen Cui, Mengzhen Zheng, Yuqin Zhao

**Affiliations:** Department of Cardiology, Institute of Cardiovascular Diseases, Xiangyang Central Hospital, Affiliated Hospital of Hubei University of Arts and Science, Xiangyang, China

**Keywords:** coronary heart disease, mental health, sleep quality, stress, social support

## Abstract

**Background:**

Globally, there is an increasing emphasis on mental health, particularly its role in the recovery of patients with coronary heart disease (CHD). Although previous studies have shown that perceived social support (PSS), mental health literacy (MHL), perceived stress and sleep quality are significantly related, the interaction mechanism remains unclear.

**Objectives:**

To explore the status of perceived stress, sleep quality, PSS and MHL in young and middle-aged patients with CHD and to test the mediating effect of PSS and MHL between perceived stress and sleep quality.

**Methods:**

A convenience sample of 183 young and middle-aged patients with CHD was enrolled between September 2023 and January 2024. Data were collected using a demographic characteristics questionnaire, the mental health literacy scale, the perceived social support scale, the Chinese perceived stress scale, and the Athens insomnia scale. Pearson correlation analysis was conducted to examine relationships between variables, while linear regression analysis was used to assess the predictive effects of perceived stress, PSS, and MHL on sleep quality. Additionally, the PROCESS 4.2 macro for SPSS was employed to explore the mediating roles of PSS and MHL in the relationship between patients’ perceived stress and sleep quality.

**Results:**

Correlation analysis revealed that perceived stress in young and middle-aged patients with CHD was negatively correlated with MHL (*r* = −0.381, *p* < 0.001) and PSS (*r* = −0.244, *p* < 0.001), but positively correlated with sleep quality (*r* = 0.349, *p* < 0.001). Additionally, the chain mediating role of PSS and MHL (3.93%) between perceived stress and sleep quality was significant.

**Conclusion:**

Perceived stress not only directly affected sleep quality in young and middle-aged patients with CHD but also could indirectly affect sleep quality through PSS and MHL.

## 1 Introduction

Coronary heart disease (CHD), the leading cause of early mortality both nationally and globally (Alexim et al., 2022), poses a significant threat to human health and has become a major public health concern worldwide.

A growing body of evidence indicates a gradual increase in the prevalence and incidence of CHD among young and middle-aged adults, primarily attributed to rising rates of smoking, hypertension, diabetes mellitus, and dyslipidemia (Zheng et al., 2020). As a distinct subgroup, young and middle-aged patients with CHD are at a pivotal stage in their lives. They often face unique psychosocial stressors. Unlike older adults. In addition to managing a chronic illness, they are in the critical period of realizing their personal values and professional achievements, and they are the main bearers of the family economy and the backbone of the society, shouldering multiple responsibilities such as taking care of their children and raising their parents (Rubin and Borden, 2012; Virani et al., 2023). However, once CHD strikes, they face more threats than other groups of people in various aspects, such as forced interruption of social activities, decreased social participation, and limited return to work. The onset of CHD can interrupt their social participation and professional roles, contributing to psychological stress. Meanwhile, patients often face varying degrees of fear of disease recurrence after experiencing chest pain (Fait et al., 2018), which can further impact their psychological distress and sleep quality (Xu et al., 2023). Compared with the general population, these patients are more susceptible to perceived stress (Gao et al., 2022) and sleep disorders (SD) (Guo, 2023), both of which are closely interrelated. Notably, perceived stress has been identified as a key predictor of poor sleep quality (Zhao et al., 2021), and is also strongly associated with the onset and progression of CHD (Gao et al., 2022; Richardson et al., 2012; Elendu et al., 2024). Furthermore, stress-induced myocardial ischemia affects a significant proportion of CHD patients, ranging from 15 to 70% (McKinnon et al., 2021).

SD is a significant risk factor for cardiovascular disease and a symptom of underlying physical or mental health issues (Grandner et al., 2016). It affects approximately 20.3 to 23.8% of young and middle-aged individuals (Zhou et al., 2020; Baldini et al., 2024). In this context, perceived social support (PSS)—defined as an individual’s emotional experience and satisfaction from feeling supported and understood by others—has been shown to alleviate stress and improve sleep quality (Zimet et al., 1990; van Schalkwijk et al., 2015). The high mortality and morbidity of CHD can exacerbate patients’ conditions. Approximately 50% of hospitalized patients experience psychological problems, which can significantly affect treatment outcomes and prognosis. Consequently, the link between mental health and the increased risk of CHD is receiving growing attention. Mental health literacy (MHL), referring to individuals’ knowledge and behaviors that promote mental health and help manage mental illness (Jiang et al., 2020), may also play a critical role. However, MHL remains relatively low among the Chinese public (Root and Caskie, 2023), which may hinder individuals from effectively addressing psychological stress. According to the main effects model of PSS, PSS directly enhances mental health and positively influences MHL, ultimately promoting healthier coping behaviors.

Although prior studies have explored the associations among perceived stress, PSS, MHL, and sleep quality, few have examined their combined effects within an integrated framework. To address this gap, the present study proposes a chain mediation model, grounded in the stress-buffering and main effect models of social support (Lam, 2024), wherein PSS and MHL sequentially mediate the relationship between perceived stress and sleep quality in young and middle-aged patients with CHD. PSS may alleviate stress by providing emotional and informational support and may also enhance MHL, which in turn facilitates adaptive coping strategies.

Based on the theoretical framework and existing evidence, the following hypotheses are proposed:

*H1*: Perceived stress will be negatively associated with sleep quality.
*H2*: PSS will mediate the relationship between perceived stress and sleep quality.
*H3*: MHL will mediate the relationship between perceived stress and sleep quality.
*H4*: PSS and MHL will function as chain mediators in the relationship between perceived stress and sleep quality.

By testing these hypotheses, this study aims to explore relationship between the four, and to provide insights for developing targeted interventions to improve sleep quality in young and middle-aged patients with CHD.

## 2 Materials and methods

### 2.1 Design and participants

This study was a cross-sectional survey. Participants were recruited between September 2023 and January 2024 through convenience sampling from the cardiovascular medicine department of a tertiary hospital in Hubei, China. Inclusion Criteria: Patients diagnosed with CHD confirmed by coronary angiography; aged 18 to 59 years; in stable condition with clear consciousness and the ability to comprehend the questionnaire accurately; willing to participate voluntarily and cooperate with the study. Exclusion Criteria: Patients with severe comorbidities that significantly affect survival, such as malignant tumors or advanced kidney diseases. Based on the cross-sectional sample size estimation method (Xu et al., 2021), the questionnaire consists of 24 items, and the sample size should be 5 to 10 times the number of questionnaire items. Accounting for a 10% invalid questionnaire rate, at least 132 valid responses should be collected. The study involving human participants was reviewed and approved by Xiangyang Central Hospital (NO: 2023128). Participants provided written informed consent to participate in this study.

### 2.2 Measurements

#### 2.2.1 Demographic characteristics

The demographic characteristics of the participants included age, gender, body mass index (BMI), education level, marital status, per capita monthly income, residence, living situation, working conditions, smoking and drinking history, comorbid chronic conditions (including hypertension, diabetes mellitus, chronic renal insufficiency, stroke, and gout), and number of stent implants.

#### 2.2.2 Mental health literacy scale (MHLS)

The Mental Health Literacy Scale (MHLS) was used to measure individuals’ levels of MHL developed by O'Connor and Casey (2015) and revised and translated by Ma (Xu et al., 2021), the scale comprises 35 items across six dimensions. The six dimensions include: recognition of common mental disorders (8 items), attitudes to improve recognition and help-seeking behaviors (14 items), knowledge of self-treatments for patients with mental disorders (4 items), knowledge of disease risks and access to treatment (4 items), Knowledge of how to seek mental health information (4 items), knowledge of common treatments (1 item). It is assessed using a 4-point or 5-point Likert scale, with 12 items reverse-scored. Total scores range from 35 to 160, with higher scores indicating higher levels of MHL. The Cronbach’s *α* for the Chinese version of the scale was 0.811, and Cronbach’s *α* for this study was 0.802.

#### 2.2.3 Perceived social support scale (PSSS)

Individuals’ PSS was measured using the Perceived Social Support Scale (PSSS), developed by Zimet et al. (1990) revised and translated by Jiang (Ying et al., 2023), which consists of 12 items. The scale uses a 7-point Likert scale, ranging from 1 (very strongly disagree) to 7 (very strongly agree). Total scores range from 12 to 84, with higher scores indicating greater PSS. A score of 12–36 indicates low PSS, 37–60 indicates moderate perceived social support, and 61–84 indicates high PSS. The Cronbach’s α for the Chinese version of the scale was 0.880, and Cronbach’s α for this study was 0.862.

#### 2.2.4 Chinese perceived stress scale (CPSS)

Mental stress was measured using the Chinese perceived stress scale (CPSS), developed by Cohen (Cerezci-Duygu et al., 2023) and revised and translated by Yang and Huang (2003). This scale consists of 14 items, divided into two dimensions: perceived distress and coping, with 7 items in each subscale. The scale uses a 5-point Likert scale, ranging from 0 (never) to 4 (always), with the perceived coping dimension reverse-scored. Total scores range from 0 to 56, with higher scores indicating higher levels of perceived stress. The Cronbach’s *α* for the Chinese version of the scale was 0.78, and the Cronbach’s α for this study was 0.815.

#### 2.2.5 The Athens insomnia scale (AIS)

Sleep quality was measured using the Athens Insomnia Scale (AIS), developed by Soldatos et al. (2000) and translated by Sun et al. (2011), which consists of 8 items. Total scores range from 0 to 24, with higher scores indicating poorer sleep quality. A score of less than 4 indicates no SD, 4–6 indicates suspected insomnia, and greater than 6 indicates insomnia. The Cronbach’s α for the Chinese version of the scale was 0.830, and the Cronbach’s α for this study was 0.926.

### 2.3 Data collection

Two trained researchers introduced the purpose and significance of this study to the patients using standardized instructions. After the patients provided informed consent, they scanned the electronic questionnaire to complete it. Researchers answered patients’ questions promptly during the questionnaire completion process. Per capita monthly income and working conditions were included in the questionnaire, and the rest of the demographic characteristics and disease information were collected through electronic medical records. Patients unable to complete the questionnaire were assisted in completing it in a question-and-answer format to exclude invalid responses. A total of 196 questionnaires were distributed, and 183 were returned, resulting in an effective recovery rate of 93.37%.

### 2.4 Statistical analysis

Statistical analysis was conducted using SPSS version 26.0. Categorical data were described by frequency and percentage (%). Continuous data were described by mean ± SD and quartiles P_50_ (P_25_, P_75_), and intergroup differences were compared using t-test, one-way ANOVA, and Kruskal-Wallis test. Besides, Harman’s single-factor test examined common method bias for all variables. Pearson’s correlation coefficient was used to analyze the correlations among the four variables, and linear regression analysis was conducted to examine the associations of perceived stress, PSS, and MHL with sleep quality. The mediation test was performed using Model 6 in PROCESS version 4.2, developed by Hayes, with confidence intervals (*CI*) by the Bootstrap method at 95% with 5,000 repeated samples. In addition, *p* < 0.05 for all tests was considered statistically significant.

## 3 Results

### 3.1 Common method bias test

Harman’s single-factor test was conducted, and the results indicated that 22 factors had eigenvalues greater than 1. The first factor explained 13.45% of the total variance, which is less than 40% (Podsakoff et al., 2003), suggesting that there is no significant common method bias in this study.

### 3.2 Comparison of characteristic data and sleep quality scores of young and middle-aged patients with CHD

A total of 183 patients with an average age of 51.48 ± 5.83 (35–59) years, participated in the study. Of these, 63 (34.43%) young and middle-aged patients with CHD had SD, including 56 males and 7 females. Other demographic characteristics are shown in Table 1.

**Table 1:** Comparison of scores on general characteristics and sleep quality in young and middle-aged patients with coronary heart disease.

Variable	*N* (%)	PSQI (Mean ± SD)	Statistical value	*p*
Age			−1.669^a^	0.097
≤45	29 (15.85)	6.10 ± 4.66		
46 ~ 59	154 (84.15)	7.79 ± 5.04		
Gender			0.833^a^	0.406
Male	156 (85.25)	7.65 ± 5.01		
Female	27 (14.75)	6.78 ± 5.00		
BMI			0.508^b^	0.602
<24	53 (28.96)	6.98 ± 4.67		
24 ~ 28	90 (49.18)	7.86 ± 5.00		
≥28	40 (21.86)	7.48 ± 5.49		
Education level				
Primary and below	28 (15.30)	7.5 (5, 16)	15.153^c^	0.002
Secondary school	64 (34.97)	5 (4, 10)		
High school/technical secondary school	48 (26.23)	5.5 (4, 12.75)		
College and above	43 (23.50)	4 (3, 6)		
Marital status			0.444^a^	0.658
Single/Divorce/Widowed	8 (4.37)	6.75 ± 3.81		
Married	175 (96.63)	7.55 ± 5.06		
Per capita monthly income			3.091^c^	0.009
<2,500	40 (21.86)	8.5 (5, 15.75)		
2,500 ~ 4,500	65 (35.52)	5 (4, 7)		
>4,500	78 (42.63)	5 (4, 11)		
Residence			1.375^a^	0.171
Village	74 (40.44)	8.14 ± 5.20		
City	109 (59.56)	7.10 ± 4.85		
Living situation			0.261^b^	0.770
Living alone	7 (3.83)	6.57 ± 3.87		
Live with spouse	59 (32.24)	7.83 ± 5.06		
Live with children and spouse	117 (63.93)	7.42 ± 5.06		
Working condition			−2.041^b^	0.043
Workless	102 (55.74)	8.19 ± 5.14		
Working/Retirement	81 (44.26)	6.68 ± 4.73		
Smoking			−1.211^a^	0.227
No	78 (42.63)	7.00 ± 4.63		
Have	105 (57.37)	7.90 ± 5.25		
Drinking			−0.044^a^	0.965
No	103 (43.72)	7.50 ± 4.92		
Have	80 (56.28)	7.54 ± 5.15		
Number of chronic diseases combined			−2.089^a^	0.038
<2	150 (81.97)	7.16 ± 4.85		
≥2	33 (18.03)	9.15 ± 5.45		
Hypertension			−1.105^a^	0.271
Have	113 (61.7)	7.84 ± 5.216		
No	70 (38.3)	7.00 ± 4.631		
Diabetes mellitus			−1.471^c^	0.141
Have	41 (22.4)	6 (4, 15)		
No	142 (77.6)	5 (4, 10)		
Stroke			−1.602^c^	0.109
Have	9 (4.9)	5 (4, 17)		
No	174 (95.1)	5 (4, 10)		
Chronic renal insufficiency			0.144^a^	0.886
Have	5 (2.7)	7.20 ± 5.59		
No	178 (97.3)	7.53 ± 5.004		
Gout			−0.698^a^	0.486
Have	4 (2.2)	9.25 ± 5.68		
No	179 (97.8)	7.48 ± 5.00		
Number of stents implanted			0.175^b^	0.913
0	74 (40.4)	7.24 ± 4.70		
1	54 (29.5)	7.54 ± 4.93		
2	39 (21.3)	7.79 ± 5.54		
≥3	16 (8.7)	8.06 ± 5.63		

^a^t-test: ^b^one-way ANOVA; ^c^Kruskal-Wallis test.

BMI, body mass index.

### 3.3 Correlation of PSS, MHL, perceived stress, and sleep quality in young and middle-aged patients with CHD

**Figure 1:**
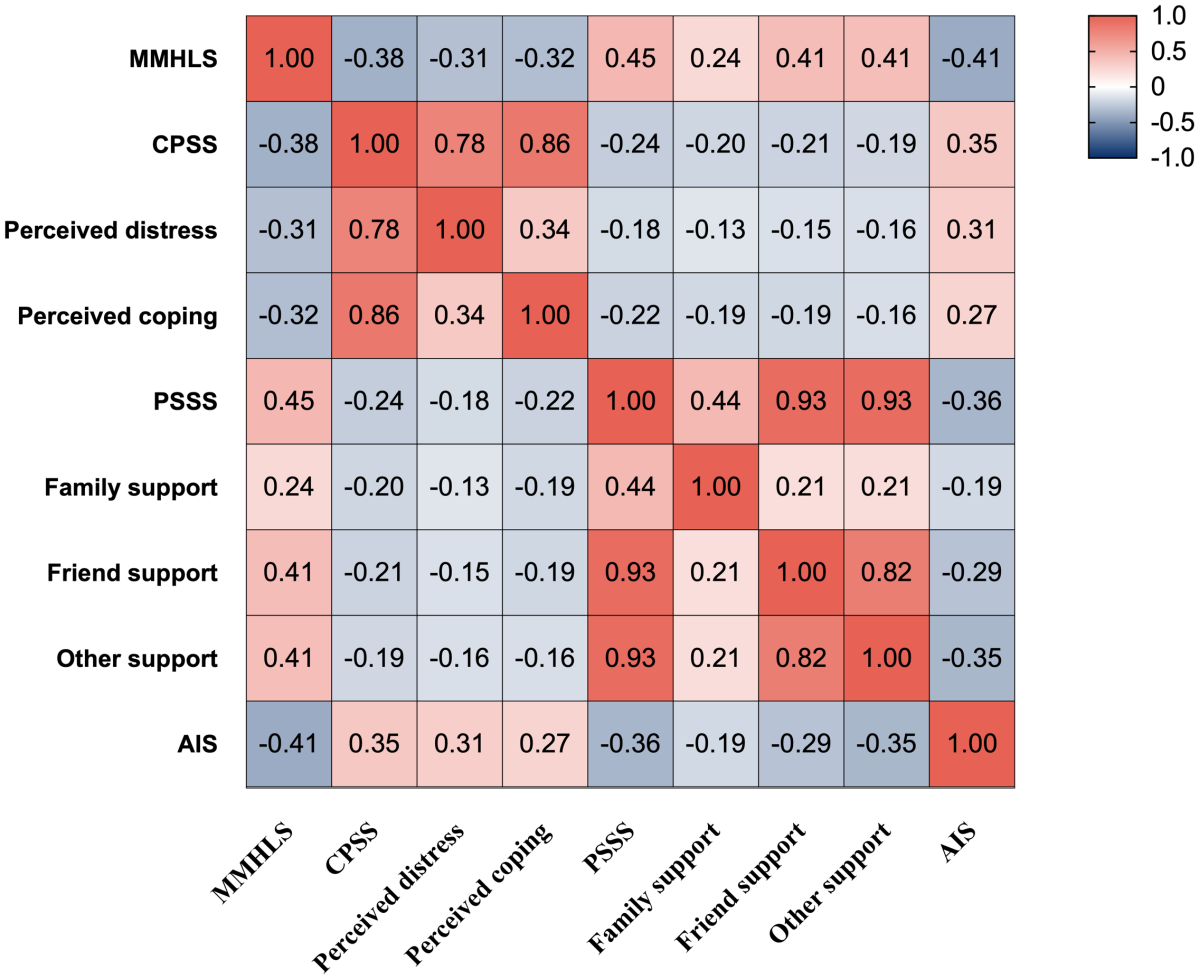
Descriptive and correlation analysis of scores for variables.

The PSS score of young and middle-aged patients with CHD was 56.80 ± 8.82, the MHL score was 95.11 ± 9.52, the perceived stress score was 29.02 ± 6.47, and the sleep quality score was 7.52 ± 5.00. This study found that perceived stress was negatively correlated with MHL and PSS (*p* < 0.001) and positively correlated with sleep quality (*p* < 0.001), as shown in Figure 1.

### 3.4 The mediating effect of PSS and MHL between perceived stress and sleep quality in young and middle-aged patients with CHD

**Figure 2:**
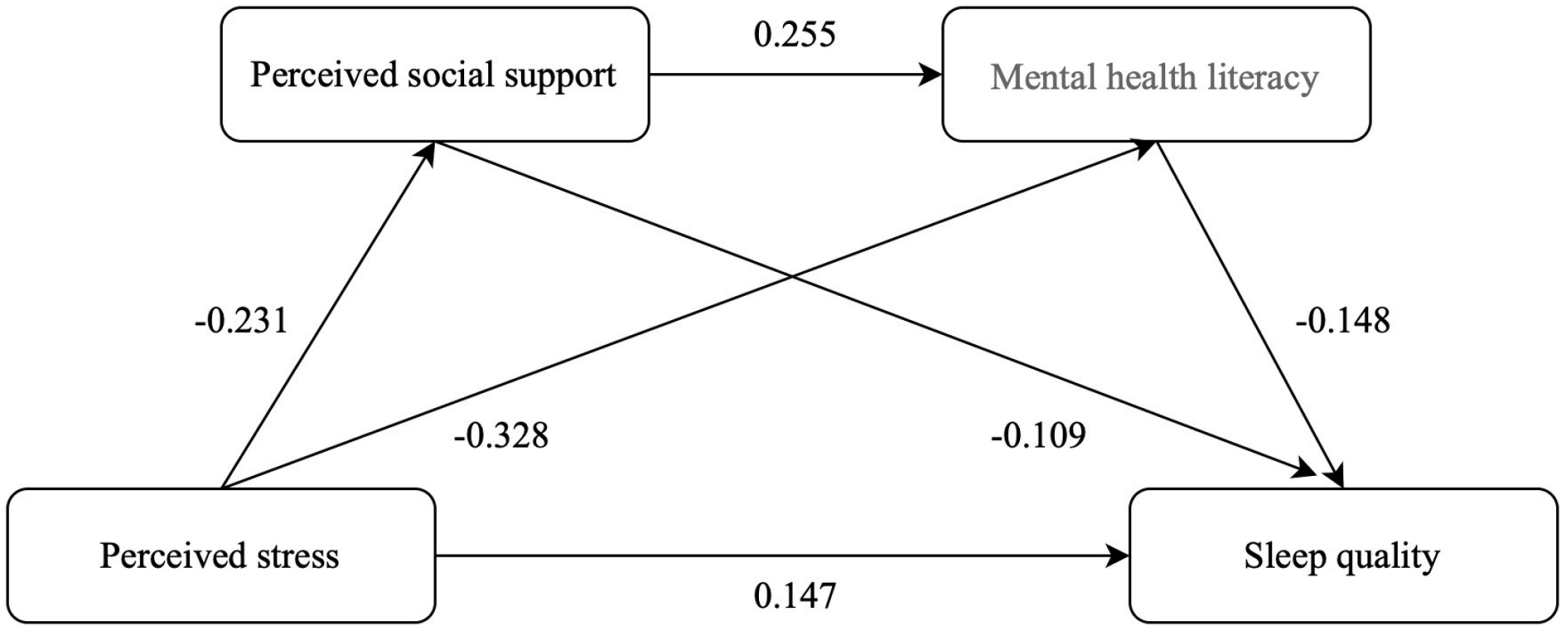
Mediating model and regression coefficient of perceived social support and mental health literacy in the relationship between perceived stress and sleep quality.

The mediating effect of PSS and MHL between perceived stress and sleep quality was examined, controlling for education level, per capita monthly income, working conditions, and the number of chronic diseases. Perceived stress and PSS were associated with MHL, while both PSS and MHL were significantly associated with sleep quality. The total indirect effect was 0.082. The 95% CI of the three mediating paths did not include 0, indicating that the mediating role was significant. The total indirect effect accounted for 35.81% of the total effect, as shown in Tables 2, 3 and Figure 2.

**Table 2:** Regression analysis of the relationship between variables in the mediation model.

Outcome variable	Predictor variables	*R*	*R^2^*	*F*	β	*t*	*p*
PSSS^a^	CPSS	0.388	0.150	6.271^**^	−0.231	−2.354	0.020
MMHLS^a^	CPSS	0.700	0.490	28.140^**^	−0.328	−3.925	<0.001
	PSSS				0.255	4.036	<0.001
AIS^a^	CPSS	0.515	0.265	9.028^**^	0.147	2.673	0.008
	PSSS				−0.109	−2.609	0.010
	MMHLS				−0.148	−3.094	<0.001

AIS, The Athens insomnia scale; CPSS, Chinese perceived stress scales; PSSS, Perceived social support scale; MMHLS, Multicomponent mental health literacy scale.

^a^Statistically significant education level, per capita monthly income, working condition and number of chronic diseases combined were included in the linear regression.

^**^p < 0.001.

**Table 3:** Analysis of the mediating role of perceived social support and mental health literacy in young and middle-aged patients with coronary heart disease.

Variable	Effect	Boot SE	Bootstrap 95%CI	Relativistic effect
Total effect	0.229	0.055	0.121 ~ 0.338	
Direct effect	0.147	0.055	0.039 ~ 0.256	64.19%
Indirect effect	0.082	0.026	0.036 ~ 0.136	35.81%
CPSS→PSSS→AIS	0.025	0.016	0.002 ~ 0.065	10.92%
CPSS→MMHLS→AIS	0.048	0.020	0.013 ~ 0.091	20.96%
CPSS→PSSS→MMHLS→AIS	0.009	0.006	0.001 ~ 0.022	3.93%

AIS, The Athens insomnia scale; CPSS, Chinese perceived stress scales; PSSS, Perceived social support scale; MMHLS, Multicomponent mental health literacy scale.

## 4 Discussion

The perceived stress score of young and middle-aged patients with CHD was 29.02 ± 6.47, indicating a high level of stress, which aligns with a previous study (Qin et al., 2021). This may be due to the fact that a diagnosis of CHD is a negative event that takes time to accept. Furthermore, in today’s highly competitive social environment, young and middle-aged individuals face multiple pressures, including interpersonal relationships, career advancement, mortgage payments, children’s education, and caregiving for the elderly. Under the dual pressures of illness and daily life demands, patients experience a greater psychological burden than the general population and are more susceptible to emotional disturbances, such as tension and anxiety, which in turn adversely affect their sleep. Therefore, healthcare professionals must remain vigilant to the psychological stress experienced by patients with CHD and consider providing psychoemotional interventions and appropriate support.

The PSS of young and middle-aged patients with CHD was 56.80 ± 8.82, indicating a medium level of stress, consistent with the findings of Du et al. (2024). Our findings support the notion that PSS acts as a protective factor by alleviating the adverse effects of perceived stress and promoting better sleep quality. During hospitalization, limited social interaction and lack of disease-related knowledge may hinder the development of support systems, specially, participants in this study were young and middle-aged patients newly diagnosed with CHD. A study (Klinger et al., 2023) has shown that young and middle-aged individuals can obtain health management information from PSS to enhance their health management behaviors. Healthcare professionals should offer targeted health education to this group and encourage participation in positive social activities, such as organizing patient exchanges and in-clinic workshops, to facilitate the acquisition of better PSS.

The MHL score of young and middle-aged patients with CHD was 95.11 ± 9.52, indicating a low level, consistent with a previous study (Jiang et al., 2021). This may be attributed to the insufficient promotion of mental health knowledge and the limited scope of psychological support in our country. Young and middle-aged patients newly diagnosed with CHD may initially experience denial or emotional resistance, reducing their willingness or capacity to engage in mental health self-management.

The sleep quality score of 7.52 ± 5.00 in young and middle-aged patients with CHD was poor, consistent with the study by Guo (2023). In addition to disease-related discomfort, stress-induced emotional disturbances such as anxiety and sadness may contribute to SD. Our study also found that males are more likely to experience SD compared to females, consistent with prior research (Wilfling et al., 2019). This gender difference may be attributed to lifestyle disparities between males and females. A study (Lee et al., 2025) showed that lifestyle factors such as obesity, lack of physical activity, smoking or alcohol dependence, as well as disease-related sleep problems like sleep apnea, are more prevalent in males. Combined with the perceived stress from social loss due to changes in life status and social roles, these factors may contribute to the increased risk of SD in young male CHD patients.

Firstly, this study showed that PSS plays an intermediary role in the link between perceived stress and sleep quality, accounting for 10.92% of the total indirect effect. Consistent with prior research (Alan et al., 2020), higher levels of PSS are associated with reduced perceived stress and better great sleep quality. PSS as a crucial external asset, feeling supported by others can help patients better cope with life disruptions caused by illness. On the other hand, prolonged exposure to high perceived stress diminishes a person’s level of PSS of the outside world and may disrupt biological rhythms essential for healthy sleep (Alan et al., 2020; Lu et al., 2023). Secondly, MHL emerged as another significant mediator, explaining 20.96% of the indirect effect. Persistent psychological tension can gradually undermine mental resilience—the ability to adapt and maintain psychological stability under adverse conditions. Sustained arousal during the day may persist into the night, disrupting emotional regulation and impairing sleep continuity. Patients with a stronger understanding of mental well-being are more capable of recognizing problems and adopting adaptive strategies to mitigate their impact (Jiang et al., 2020). When faced with elevated stress, those with higher psychological awareness are better equipped to reframe challenges and regain emotional balance, thereby improving sleep quality. Additionally, although the chain mediation effect accounted for only 3.93% of the total effect, it remained statistically significant, indicating a subtle yet meaningful pathway. In practice, this finding suggests that even small improvements in PSS and MHL could jointly effect sleep quality in patients with CHD. Previous studies have shown that perceived stress typically exacerbates sleep problems, creating a negative feedback loop that worsens emotions, health, and sleep quality (Wang et al., 2016). According to the social support-buffering model, support systems not only offer protective but also foster a more proactive approach to mental self-care (Jing et al., 2023). When faced with illness, patients must manage the demands of sudden life changes and require age-appropriate information and resources to address new challenges (Kruszecka-Krowka et al., 2025). For patients dealing with CHD in their prime working years, PSS serve as essential pathways for information, empathy, and tangible help, through various social relationships (e.g., family and friends) receiving emotional support, informational support (e.g., disease advice), other forms of support (e.g., financial and online support). Strengthening these relational resources, while simultaneously improving individuals’ mental health competence, may offer a comprehensive approach to alleviating stress-related sleep issues. Therefore, young and middle-aged individuals with CHD should actively seek effective social support to enhance MHL, thereby buffering the adverse effects of perceived stress on sleep quality.

This study has several limitations. First, due to limitations in time, resources, and sample size, this study included only young and middle-aged patients with CHD from Xiangyang. Future research should increase the sample size through multi-center surveys to enhance the generalizability of the results. Second, this study was limited by its cross-sectional design, which lacked longitudinal data. Additionally, inter-individual variability may have affected the accuracy of the conclusions. Third, the use of self-reported questionnaires may introduce subjectivity, and future studies could employ objective tools to mitigate this limitation. Additionally, convenience sampling was employed due to the accessibility of eligible participants during routine hospital visits. While this approach allowed for timely data collection, it may limit the representativeness of the sample and affect the generalizability of the findings. Future studies should consider using randomized or stratified sampling to enhance external validity.

## 5 Conclusion

This study demonstrated that PSS and MHL influence perceived stress and sleep quality, mediating their relationship. Young and middle-aged patients with CHD exhibit poor sleep quality; therefore, healthcare providers should monitor their sleep patterns, prioritize early psychological assessments, and implement appropriate interventions to improve sleep quality.

## Data Availability

The raw data supporting the conclusions of this article will be made available by the authors, without undue reservation.
